# 1153. Safety and Potential Efficacy of Exosomes Overexpressing CD24 (EXO-CD24) for the Prevention of Clinical Deterioration in Patients with Moderate or Severe COVID-19: A Phase II, Randomized, Single-blinded Study.

**DOI:** 10.1093/ofid/ofac492.991

**Published:** 2022-12-15

**Authors:** Ioannis Grigoropoulos, Georgios Tsioulos, Artemis Kastrissianakis, Shiran Shapira, Nadir Arber, Garyfallia Poulakou, Konstantinos Syrigos, Vasiliki Rapti, Ioannis Xynogalas, Konstantinos Leontis, Vasileios Ntousopoulos, Vissaria Sakka, Mina Gaga, Zafeiris Sardelis, Andreas Fotiadis, Lamprini Vlassi, Chrysoula Kontogianni, Anastasia Levounets, Maria Tsakona, Athina Savva, Dimitra Kavatha, Dimitrios Boumpas, Anastasia Antoniadou, Sotirios Tsiodras

**Affiliations:** 4th Department of Internal Medicine, University General Hospital Attikon, Medical School, National and Kapodistrian University of Athens, 12462 Athens, Greece, Athens, Attiki, Greece; 4th Department of Internal Medicine, University General Hospital Attikon, Medical School, National and Kapodistrian University of Athens, 12462 Athens, Greece, Athens, Attiki, Greece; 4th Department of Internal Medicine, University General Hospital Attikon, Medical School, National and Kapodistrian University of Athens, 12462 Athens, Greece, Athens, Attiki, Greece; Integrated Cancer Prevention Center, Tel Aviv Medical Center, Tel Aviv 6423906, Israel, Tel Aviv, Tel Aviv, Israel; Integrated Cancer Prevention Center, Tel Aviv Medical Center, Tel Aviv 6423906, Israel, Tel Aviv, Tel Aviv, Israel; 3rd Department of Internal Medicine, National and Kapodistrian University of Athens, Medical School, Sotiria General Hospital, Athens, Attiki, Greece; 3rd Department of Internal Medicine, National and Kapodistrian University of Athens, Medical School, Sotiria General Hospital, Athens, Attiki, Greece; 3rd Department of Internal Medicine, National and Kapodistrian University of Athens, Medical School, Sotiria General Hospital, Athens, Attiki, Greece; 3rd Department of Internal Medicine, National and Kapodistrian University of Athens, Medical School, Sotiria General Hospital, Athens, Attiki, Greece; 3rd Department of Internal Medicine, National and Kapodistrian University of Athens, Medical School, Sotiria General Hospital, Athens, Attiki, Greece; 3rd Department of Internal Medicine, National and Kapodistrian University of Athens, Medical School, Sotiria General Hospital, Athens, Attiki, Greece; 3rd Department of Internal Medicine, National and Kapodistrian University of Athens, Medical School, Sotiria General Hospital, Athens, Attiki, Greece; 7th Respiratory Medicine Dept, Athens Chest Hospital "Sotiria", Athens, Attiki, Greece; 7th Respiratory Medicine Dept, Athens Chest Hospital "Sotiria", Athens, Attiki, Greece; 7th Respiratory Medicine Dept, Athens Chest Hospital "Sotiria", Athens, Attiki, Greece; 7th Respiratory Medicine Dept, Athens Chest Hospital "Sotiria", Athens, Attiki, Greece; 7th Respiratory Medicine Dept, Athens Chest Hospital "Sotiria", Athens, Attiki, Greece; 7th Respiratory Medicine Dept, Athens Chest Hospital "Sotiria", Athens, Attiki, Greece; 4th Department of Internal Medicine, University General Hospital Attikon, Medical School, National and Kapodistrian University of Athens, 12462 Athens, Greece, Athens, Attiki, Greece; 4th Department of Internal Medicine, University General Hospital Attikon, Medical School, National and Kapodistrian University of Athens, 12462 Athens, Greece, Athens, Attiki, Greece; 4th Department of Internal Medicine, University General Hospital Attikon, Medical School, National and Kapodistrian University of Athens, 12462 Athens, Greece, Athens, Attiki, Greece; 4th Department of Internal Medicine, University General Hospital Attikon, Medical School, National and Kapodistrian University of Athens, 12462 Athens, Greece, Athens, Attiki, Greece; 4th Department of Internal Medicine, University General Hospital Attikon, Medical School, National and Kapodistrian University of Athens, 12462 Athens, Greece, Athens, Attiki, Greece; 4th Department of Internal Medicine, University General Hospital Attikon, Medical School, National and Kapodistrian University of Athens, 12462 Athens, Greece, Athens, Attiki, Greece

## Abstract

**Background:**

EXO-CD24 is a novel inhaled drug of exosomes displaying CD24, a protein with anti-inflammatory properties. We evaluated the safety and potential efficacy of EXO-CD24, in a phase II, randomized, single-blinded clinical trial of EXO-CD24 in hospitalized patients with moderate or severe COVID-19, following the preliminary safety and efficacy results of a phase 1 study (ClinicalTrials.gov: NCT04747574).

**Methods:**

Two tertiary care hospitals in Athens, Greece participated. Patients received either 10^9^ or 10^10^ exosome particles per dose, once per day for 5 days and were followed for 28 days. Safety and efficacy measures (including respiratory rate < 23 b/min and pulse oximetry SpO_2_≥ 94% on room air, oxygen need and levels of inflammatory biomarkers i.e. CRP, LDH, ferritin, fibrinogen and d-dimers) were compared between groups at days 3, 5 and 7. A separate analysis was conducted comparing the clinical course of treated patients with that of a control cohort (n=70 patients) matched by propensity scoring out of a similar period hospitalized cohort (n=202) that did not participate in the study.

**Results:**

Between June 9th and August 3rd 2021, 91 patients underwent randomization: 45 in group A and 46 in group B (10^9^*vs.* 10^10^ exosome particles per dose). Mean age was 49.4 (± 13.2) years and 74.4% were male. Mean time from symptom onset to randomization was 8 days. Improvement in respiratory rate and pulse oximetry was noted in 72 out of 86 (83.7%) and 55 out of 86 (64%) analyzed patients. Day 7 inflammatory indices levels dropped at least 50% from baseline admission values in 72 out of 86 (82.8%) analyzed patients (p< 0.001). No treatment-related adverse events were reported. Comparison with the propensity score matched group showed statistically significant differences in the same parameters (p≤ 0.01 for all comparisons).

**Conclusion:**

Our results suggest safety and potential efficacy of EXO-CD24 on clinical and laboratory parameters of moderate or severe COVID-19, that deserve further investigation in a phase 3 study. (Funded by Athens Medical Society. ClinicalTrials.gov: NCT04902183, EU Clinical Trials Register EudraCT Number 2021-002184-22).

**Disclosures:**

**All Authors**: No reported disclosures.

